# BmncRNAdb: a comprehensive database of non-coding RNAs in the silkworm, *Bombyx mori*

**DOI:** 10.1186/s12859-016-1251-y

**Published:** 2016-09-13

**Authors:** Qiu-Zhong Zhou, Bindan Zhang, Quan-You Yu, Ze Zhang

**Affiliations:** 1 Laboratory of Evolutionary and Functional Genomics, School of Life Sciences, Chongqing University, Huxi Campus, No. 55 Daxuecheng South Rd., Shapingba, Chongqing, 401331 China; 2 School of Economics and Business Administration, Chongqing University, Campus A, No. 174 Shazheng Rd., Shapingba, Chongqing, 400044 China

**Keywords:** Silkworm, Long non-coding RNAs, RNA-seq, BmncRNAdb

## Abstract

**Background:**

Long non-coding RNAs (lncRNAs) may play critical roles in a wide range of developmental processes of higher organisms. Recently, lncRNAs have been widely identified across eukaryotes and many databases of lncRNAs have been developed for human, mouse, fruit fly, etc. However, there is rare information about them in the only completely domesticated insect, silkworm (*Bombyx mori*).

**Description:**

In this study, we systematically scanned lncRNAs using the available silkworm RNA-seq data and public unigenes. Finally, we identified and collected 6281 lncRNAs in the silkworm. Besides, we also collected 1986 microRNAs (miRNAs) from previous studies. Then, we organized them into a comprehensive and web-based database, BmncRNAdb. This database offers a user-friendly interface for data browse and online analysis as well as the three online tools for users to predict the target genes of lncRNA or miRNA.

**Conclusions:**

We have systematically identified and collected the silkworm lncRNAs and constructed a comprehensive database of the silkworm lncRNAs and miRNAs. This work gives a glimpse into lncRNAs of the silkworm and lays foundations for the ncRNAs study of the silkworm and other insects in the future. The BmncRNAdb is freely available at http://gene.cqu.edu.cn/BmncRNAdb/index.php.

**Electronic supplementary material:**

The online version of this article (doi:10.1186/s12859-016-1251-y) contains supplementary material, which is available to authorized users.

## Background

The ENCODE project estimates that 62–75 % of the human genome are transcribed, but only 2 % of the transcripts can be translated to proteins [1, 2]. The GENCODE 22 release contains 19,814 protein-coding genes, 15,900 long non-coding RNA genes and 9894 small non-coding RNA genes [3]. These suggest that non-coding RNAs (ncRNAs) constitute a large fraction of the eukaryote transcriptome [4, 5].

Long non-coding RNAs (lncRNAs) are transcripts of DNA that are usually considered to be > = 200 nt (nucleotide) and do not have apparent coding capacity [6–10]. LncRNAs are widely present in the eukaryotic genomes [4, 11]. In the postgenomic era, since the development and application of next-generation sequencing technologies, a large number of long non-coding RNAs have been identified in different species (e.g. human [12], mouse [13], fruit fly [14], etc.). Although the functions of most lncRNAs are still unclear, more and more evidence has proven that they play critical roles in various biological processes including cellular differentiation [15], epigenetics [16], transcriptional regulation [17] and immune response [18]. For example, in the placental mammals, *Xist* (X-inactive specific transcript) is a long non-coding RNA on the X-chromosome and takes part in inactivation of X-chromosome during the early developmental process of female embryo [8, 19]. In addition, thousands of lncRNAs have been reported in the insects and some of them show important roles in the life events of insects [14, 20–25]. *Acal* acts as a novel negative dorsal closure regulator during *Drosophila* embryogenesis and *Lnccov1* is involved in the autophagic cell death of ovarioles in *Apis mellifera* [26, 27]. Therefore, lncRNAs are important functional elements in the genomes of higher organisms.

The domesticated silkworm, *Bombyx mori*, is one of important model organisms for Lepidoptera, more and more transcrptomic resources are available for the silkworm. The ncRNAs, especially microRNAs (miRNAs) were identified in the silkworm by Solexa sequencing [28]. In addition, the miRNAs are also reported that may take part in the fibroin synthesis and fibroin transport in the domesticated silkworm [29]. As one important member of the ncRNAs, lncRNAs also play key roles in the silkworm. The first silkworm lncRNA, *Fben-1* (female-brain expressed noncoding RNA-1) was identified in female-brain and may be involved in sexually dimorphic brain functions [30]. Although 11,810 silkworm lncRNAs are identified in different tissues with the loose standard, the loose threshold may lead to high false positive rate for lncRNA identification. Thus, it is still necessary to systematically identify the lncRNAs in the silkworm with more RNA-seq data and more stringent pipeline [30, 31].

Moreover, many databases on the information of lncRNA have been developed such as NONCODE, lncRNAdb, LncRBase, DeepBase [32–35], but the information of the silkworm lncRNA is almost blank in the present lncRNA databases [32–34, 36–39]. Currently, miRBase and microrna are two large databases containing miRNA information, however, the information of the silkworm miRNAs in the miRBase is rare and redundancy [40, 41]. Thus, in this study, we used a comprehensive approach to identify lncRNAs in the silkworm with all newly released RNA-seq data in the SRA (Sequence Read Archive) database and the unigene data [7, 13, 14, 25, 42–48]. The identified lncRNAs are organized into a database for user browser. In order to offer more information about the silkwrom ncRNAs, the available silkworm miRNAs and previously reported lncRNAs are also added to the database [28, 29, 40]. The database can be accessed at the website http://gene.cqu.edu.cn/BmncRNAdb/index.php.

## Construction and content

### Database architecture

**Fig. 1:**
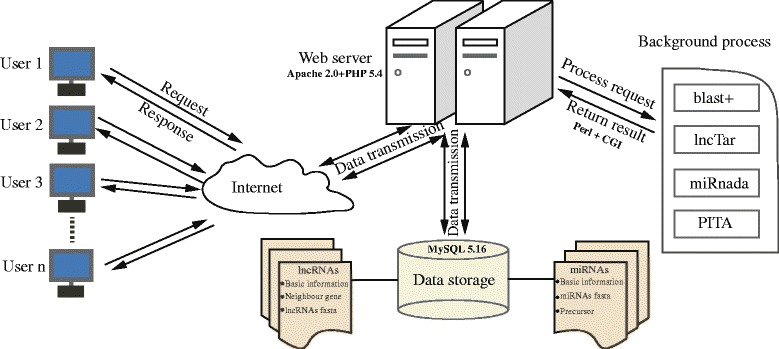
BmncRNAdb database scheme

The BmncRNAdb database implementation is based on the Gentoo Linux system with the tools of Apache 2.0 [49], PHP 5.4 (Personal home page Hypertext Preprocessor) [50], MySQL 5.16 [51], and Perl 5.12 [52]. The database architecture is illustrated in Fig. 1. Apache + PHP processes the user request and responds to user by the web browser. MySQL is used to create data model and data storage. The Perl script calls the background program to execute server request and returns the results to server by the CGI (Common Gateway Interface). Next, the web server will send the results of background program to BmncRNAdb user by the internet.

### Data sets

New version of the silkworm genome sequence was downloaded from the silkworm genome database, SilkDB v2.0 [53]. The silkworm protein-coding genes were retrieved from Ensembl database (http://metazoa.ensembl.org/) [54]. All the silkworm RNA-seq data were downloaded from NCBI (National Center for Biotechnology Information) SRA databases (Additional file 1: Table S1) (http://www.ncbi.nlm.nih.gov/sra) [55–65]. The silkworm unigenes were downloaded from NCBI UniGene database [66]. Non-redundant protein (nr) sequences were also obtained from NCBI database [66]. A comprehensive protein database, Uniref100, was downloaded from UniProt databases (http://www.uniprot.org/) [67]. The current released (Pfam 28) Pfam-A and Pfam-B were obtained from EBI ftp website (ftp://ftp.ebi.ac.uk/) [68].

### Genome-wide identification of lncRNAs in the silkworm

**Fig. 2:**
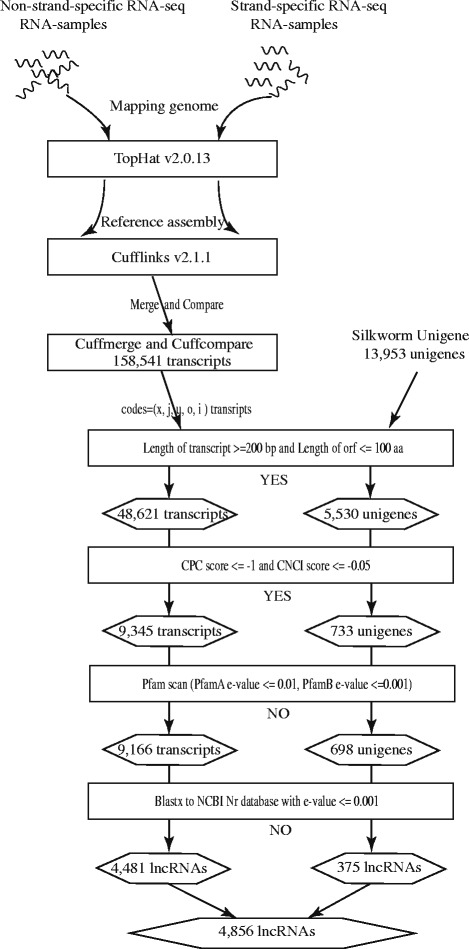
Flowchart of lncRNAs identification in the silkworm. Left pipeline means the identification of lncRNAs from RNA-seq, and the right pipeline means the identification of lncRNAs from unigenes

Two types of data from the silkworm were used for identification of the silkworm lncRNAs. The first is the silkworm RNA-seq data. Forty-one RNA-seq datasets were published by other research groups before January 15(th), 2015 and four RNA-seq datasets were produced by our laboratory (Additional file 1: Table S1) [55–65]. All the RNA-seq data are used to reconstruct the silkworm transcriptome using the software Tophat v2.0.13 and Cufflinks v2.1.1 [7, 25, 42, 43, 45, 46, 48, 69, 70]. The second is the silkworm unigenes. The unigene transcripts were assembled from EST (Expressed Sequence Tag) and some lncRNAs are also contained in the unigene transcripts [43]. Thus, the transcripts assembled from RNA-seq data and unigenes are used to identify lncRNAs in this study. The whole workflow to identify the silkworm lncRNAs is shown in Fig. 2.

#### RNA-seq short-reads assembly

QC (quality control) Toolkit of NGS (Next-Generation Sequencing) is used to control the reads quality of forty-five RNA-seq datasets [71]. High-quality RNA-seq reads are considered as clean reads data. The clean reads data were mapped to the newly assembled silkworm genome sequence with TopHat v2.0.13 [69]. Mapped reads for each sample were assembled using Cufflinks v2.1.1 with the protein-coding gene annotations separately [13, 69, 70]. All the sample assemblies are integrated into a merged assembly by Cuffmerge v2.1.1. We then used Cuffcompare v2.1.1 to generate different categories of the transcripts for the merged assembly [25, 43]. After that, 158,541 transcripts were generated from the transcriptome assembly. The five categories of the transcripts are retained including falling entirely within a reference intron (code=‘i’), sharing at least one splice junction with a reference transcript (code=‘j’), generic exonic overlap with a reference transcript (code=‘o’), unknown or intergenic transcript (code=‘u’) and exonic overlap with reference on the opposite strand (code=‘x’) [13, 69]. These five categories of the transcripts and the silkworm unigenes are used to identify lncRNAs in the next step.

#### Protein-coding transcripts exclusion

LncRNAs are usually considered to have length > =200 bp and ORFs (open reading frame) < = 100 aa (amino acids) [7, 42, 43, 70]. The assembled transcripts and unigene transcripts with the length < 200 bp or ORFs > 100 aa are excluded by the Perl Script, respectively. The retained 48,621 transcripts and 5530 unigenes are evaluated to the protein-coding potentiality for each transcript by the two tools, CPC (Coding Potential Calculator) and CNCI (Coding-Non-Coding Index) [42, 43, 70, 72–76]. In general, transcripts with protein-coding score < 0 in the CPC or CNCI are regarded as non-coding potentiality [72, 73]. The CPC and CNCI can be complementary and improve the positive rate for lncRNA identification [72, 73]. Thus, we used two tools (CPC and CNCI) and set the protein-coding score −1 as threshold in the CPC and −0.05 as threshold in the CNCI [42, 43]. Only those transcripts have CPC score < = −1 and CNCI score < = −0.05 are retained. The retained 9345 transcripts and 733 unigenes are translated into the corresponding proteins by six frame translation and then the proteins were used to search against Pfam-A and Pfam-B databases. Transcripts that have significant hits against Pfam-A and Pfam-B will be removed [10]. At last, the blastx searches against NCBI Non-redundant protein (Nr) databases with the option e-value 0.001 were performed using retained transcripts [48, 77]. Transcripts that have a hit with Nr protein sequences were deleted in this process. In the end, 4856 lncRNAs were identified from the silkworm RNA-seq and unigenes (Fig. 2). The 95.65 % of lncRNAs belong to the ‘u’ (Unknown, intergenic transcript) category (Table 1). Moreover, in order to reduce the false positive rate for lncRNAs, 11,810 previously reported lncRNAs were re-identified by our stringent pipeline [31] and 1565 high-quality lncRNAs were retained, suggesting that the false positive rate for identification of the silkworm lncRNAs in previous study may be much higher. After removing the redundancy, 6821 lncRNAs were recorded in the BmncRNAdb database. A proven previously lncRNA, *Fben-1,* is identified by our pipeline. This shows the reliability of our pipeline.

**Table 1:** The summary of the silkworm lncRNAs identified by RNA-seq

Class code	Transcript number	Percentage	Description
j	16	0.36 %	At least one splice junction is shared with a reference transcript
o	26	0.58 %	Generic exonic overlap with a reference transcript
u	4286	95.65 %	Unknown, intergenic transcript
x	151	3.37 %	Exonic overlap with reference on the opposite strand
i	2	0.04 %	A transfrag falling entirely within a reference intron
total	4481	100 %	

### Characteristics of the silkworm lncRNAs

**Fig. 3:**
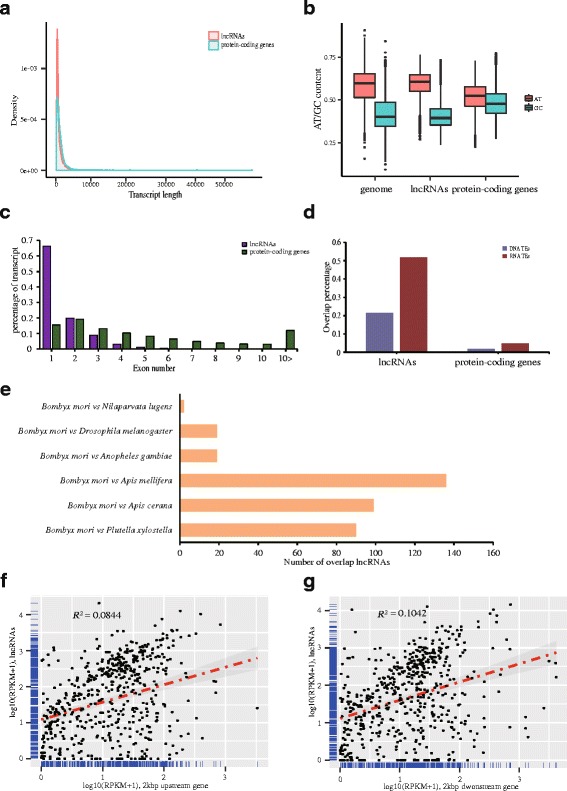
Characteristics of silkworm lncRNAs. **a** Transcript length distribution in the lncRNAs and protein-coding genes. **b** AT/GC content among the silkworm genome, lncRNAs, and protein-coding genes. **c** Transcript exon number distribution. **d** Percentage of transcripts with at least 15 nt overlapping with transposable elements. **e** Sequence conservation between the silkworm (*Bombyx mori*) lncRNAs and other insects’ lncRNAs (*Plutella xylostella*, *Apis cerana*, *Apis mellifera*, *Anopheles gambiae*, *Drosophila melanogaster*, *Nilaparvata lugens*). The x axis is the number of silkworm lncRNAs that overlap with other insects’ lncRNAs at least 15 bp. **f** Expression scatter diagram in the lncRNAs and 2 kbp upstream protein-coding genes (Spearman test). **g** Expression scatter diagram in the lncRNAs and 2 kbp downstream protein-coding genes (Spearman test)

We surveyed the comprehensive characteristics of the silkworm lncRNAs including the length distribution, GC content, exon number distribution, link with transposable elements, sequence conservation and correlation with neighbor protein genes (Fig. 3). The silkworm lncRNAs have shorter transcript length than the protein-coding genes (Fig. 3a). The lncRNAs also have lower GC content and less exon number than the protein-coding genes (Fig. 3b and c). However, the lncRNAs have a large degree of overlap of transposable elements in the silkworm (Fig. 3d). The similar results were also reported in the previous studies [31, 70, 78]. The silkworm lncRNA that overlaps with other insect lncRNAs at least 15 bp is defined as sequence conservation [78]. Based on the standard, 136 silkworm lncRNAs show sequence conservation with the *Apis mellifera* (Hymenoptera) lncRNAs, the highest sequence conservation (Fig. 3e). And the silkworm lncRNAs also have relatively high sequence conservation with the *Plutella xylostella* (Lepidoptera) and *Apis cerana* (Hymenoptera) lncRNAs. However, the silkworm lncRNAs have low sequence conservation with the *Drosophila melanogaster*, *Anopheles gambiae and Nilaparvata lugens* lncRNAs. Furthermore, the expressions of the genes within 2 kbp neighbor regions (2 kbp upstream and 2 kbp downstream) of the putative silkworm lncRNAs are not significantly correlated with the expressions of lncRNAs (Spearman test) (Fig. 3f and g).

### Collection of microRNAs in the silkworm

The silkworm microRNAs were comprehensively identified in the whole body, anterior or middle and posterior silk glands by next generation sequencing technology [28, 29]. The datasets of the silkworm miRNAs were collected from miRBase and previous studies [28, 29, 40]. All miRNAs are compared by sequence pair-wise to remove redundancy and manual correction [79]. The formats of miRNAs are unified by the Perl Scripts.

## Utility and discussion

**Fig. 4:**
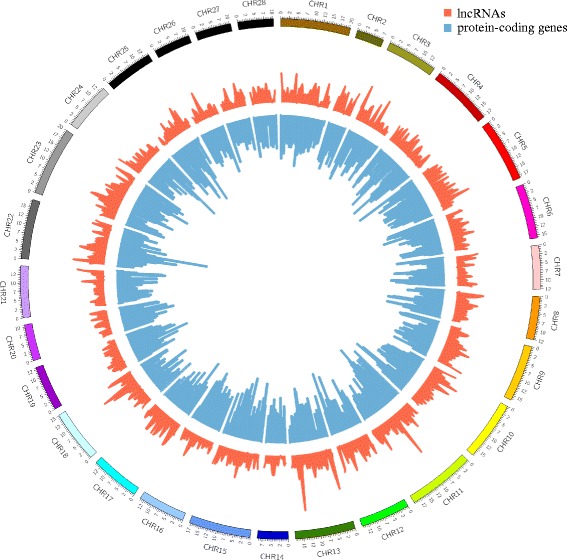
Distribution of lncRNAs and protein-coding genes on the 28 silkworm chromosomes. The abundance of lncRNAs in physical bins of 500 kb for each chromosome. The *red color* represent lncRNAs and *blue color* represent protein-coding genes

Using the pipeline in Fig. 2, we identified and collected 6281 lncRNAs. About 58.67 % of lncRNAs can be located on the silkworm chromosomes and the rest lncRNAs are located in the scaffolds that cannot be mapped to the silkworm chromosomes. All the 28 chromosomes harbored lncRNAs. Interestingly, the chromosomal distribution of the lncRNAs is not significantly correlated with the protein-coding genes (Spearman *r* = 0.017, *P*-value = 0.62) (Fig. 4). This is consistent with the observation in the lncRNAs of human [12]. Moreover, we also collected 1986 miRNAs from previous studies and public databases [28, 29, 40]. In the end, we organized these silkworm lncRNAs and miRNAs into the BmncRNAdb database (http://gene.cqu.edu.cn/BmncRNAdb/index.php). The database contains six functional sections, data browse, keywords search, Blast alignment, lncRNA target gene discovery, miRNA target gene discovery and data download.

### Data browse

**Fig. 5:**
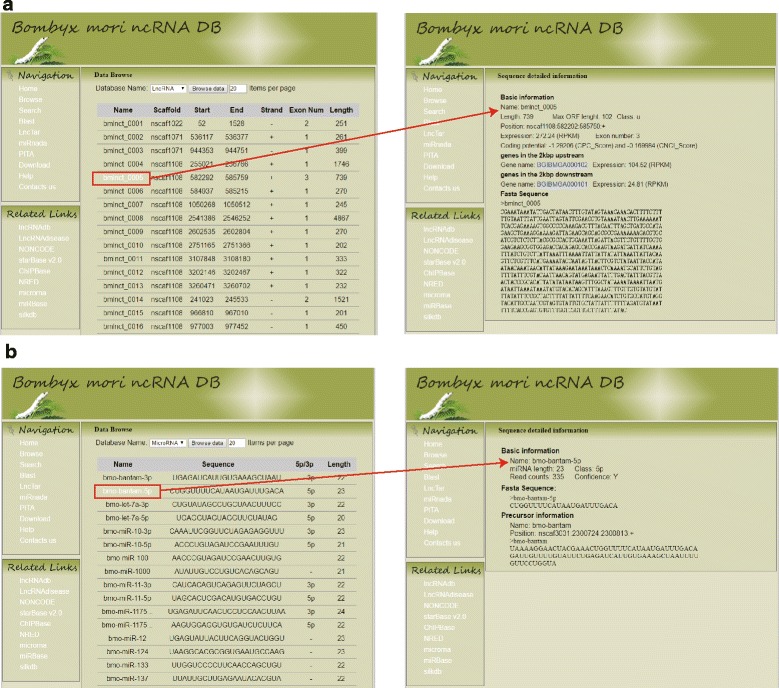
Data browse of the BmncRNAdb database. **a** The browsing interface of lncRNAs. All the silkworm lncRNAs were stored in the BmncRNAdb. Users can browse the detailed information of lncRNA by the name. **b** Data browse of miRNA. Users can get information of miRNA including basic information, fasta sequence and precursor by choosing different miRNA name

In the left navigation, clicking the ‘Browse’, users can browse the information of lncRNAs including lncRNA name, scaffold, start position, end position, exon number and length (Fig. 5a). By clicking the lncRNA name, users can obtain the detail information about the lncRNAs such as the expression, max ORF length, coding potential score, neighbor genes and fasta sequence. Moreover, clicking the names of neighbor genes, users will obtain the corresponding genome annotation information. If users want to browse the information of miRNA including miRNA name, miRNA sequence, 5p/3p class, miRNA length, they can choose the miRNA database and then click the ‘Browse data’ (Fig. 5b). By clicking the miRNA name, users can obtain the miRNA information such as miRNA length, reads count, confidence, fasta sequence and precursor information. In the search functional section, users can use keywords to search for lncRNA or miRNA in the BmncRNAdb to find the interesting entries. Although some databases (NONCODE, lncRNAdb, LncRBase, deepBase, etc.) also offer data browse for the lncRNAs, the information is mainly for human, mouse, fruit fly, etc. [32–35]. The BmncRNAdb provides not only the information for the silkworm lncRNAs but also for the lncRNAs neighbor genes and the silkworm miRNAs.

### Online analysis tools

**Fig. 6:**
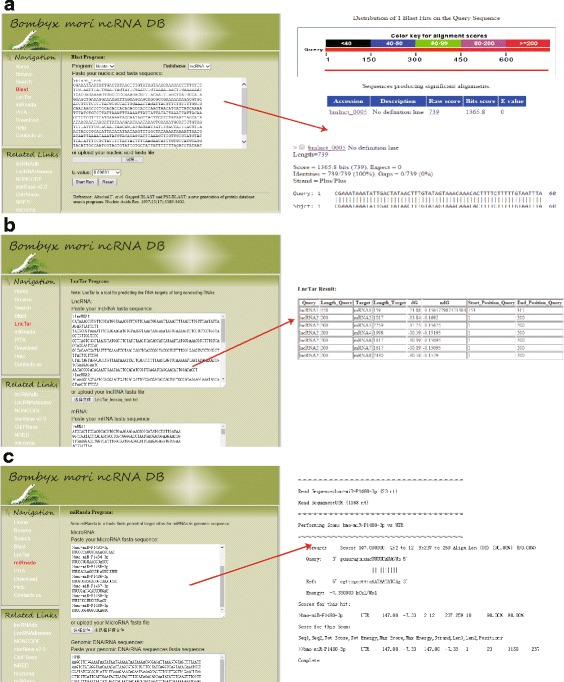
User-friendly online tools in the BmncRNAdb database. **a** Online blast program and visual output in the BmncRNAdb. Users can run blast against the silkworm lncRNA by submitting the sequence in fasta format. **b** Online predicting target gene of lncRNA interface and the results in tabular form. **c** Online predicting target gene of miRNA interface and detailed output by miRnada

The online analysis tools about lncRNAs and miRNAs are provided in the BmncRNAdb to facilitate functional research of lncRNAs and miRNAs. Four user-friendly online analysis tools are available for users including Blast + [80], LncTar [81], miRanda [41, 82] and PITA [83]. In the Blast functional section, users can submit their nucleotide sequences (fasta format) to the BmncRNAdb and quickly do search against the silkworm lncRNAs by blastn or tblastx (Fig. 6a). In the blast results, the information including the distribution of blast hit, hit score and E-value is shown. Furthermore, user can find the target sites of an lncRNA by the LncTar functional section. It is well helpful for users to find the target genes of an lncRNA by the lncRNA–mRNA interactions and free energy between lncRNA and mRNA [81]. When users run the LncTar, two types of nucleotide sequences including sequences of lncRNA and mRNA must be submitted to BmncRNAdb. An example generated by LncTar is shown in the Fig. 6b. The results will output the approximate binding free energy (dG), normalized dG (ndG) and interacted position. Like lncRNA, users can also find the target genes of a miRNA in the miRnada functional section by submitting their miRNA and DNA/RNA nucleotide sequences at the same time. An example for finding the target genes of a miRNA is shown in Fig. 6c. The score, energy and position between miRNA and DNA/RNA are shown in the miRanda result. In addition, BmncRNAdb also offers another online tool to find the target genes of a miRNA in the PITA functional section. The usage of PITA is very similar to the miRnada. All the online analysis tools are not only for the silkworm, but also can be used in other species. More help about the online tools is in the help section.

BmncRNAdb offers the download section for users to obtain all the silkworm lncRNA sequences, miRNA sequences and example data. In the help section, a guide manual is shown to help the users to learn how to better use the BmncRNAdb for their own research. In addition, under the left navigation, several useful or famous database resources about ncRNAs are collected in the BmncRNAdb related links. Our group will continue to collect more information on the silkworm ncRNAs and add more useful online tools about the functional research of ncRNAs to the BmncRNAdb in the future.

## Conclusions

We have systematically identified and collected 6281 silkworm lncRNAs using the RNA-seq data and unigenes. We also collected 1986 silkworm miRNAs that were predicted by NGS. Integrating these lncRNAs and miRNAs data, we have constructed a comprehensive lncRNAs and miRNAs database (BmncRNAdb) for the silkworm (*Bombyx mori*). Through the BmncRNAdb database, users can browse and search for the detail information of lncRNAs and miRNAs in the silkworm. In addition, this database provides three online tools for users to find the target genes of an lncRNA and miRNA. BmncRNAdb will facilitate the ncRNA research of the silkworm and other insects in the future. Moreover, the availability of the complete set of lncRNAs from the silkworm will improve the comparative and evolutionary analyses of lncRNAs among different Lepidoptera or other insect species.

## Availability and requirements

**Database:** BmncRNAdb

**Database homepage:**http://gene.cqu.edu.cn/BmncRNAdb/index.php

**Operating system(s):** Linux

**Programming language:** PHP, CGI, JavaScript, Perl

**Other requirements:** MySQL, Apache

The database is freely available without restrictions for use by academics and non-commercial researches. Inquiries concerning the database may be directed to zezhang@cqu.edu.cn or huaxia2033@126.com.

## Additional file

Additional file 1: Table S1. The detail information of RNA-seq datasets. All the samples used to the identification of the silkworm lncRNAs. (DOC 44 kb)

## Abbreviations

Aa: Amino acids
CGI: Common gateway interface
CNCI: Coding-non-coding index
CPC: Coding potential calculator
dG: Binding free energy
EST: Expressed sequence tag
*Fben-1*: Female-brain expressed noncoding RNA-1
lncRNAs: Long non-coding RNAs
miRNAs: microRNAs
NCBI: National Center for Biotechnology Information
ncRNAs: Non-coding RNAs
ndG: Normalized dG
NGS: Next-generation sequencing
Nr: Non-redundant protein
nt: Nucleotide
ORF: Open reading frame
PHP: Personal home page hypertext preprocessor
QC: Quality control
SRA: Sequence read archive
*Xist*: X-inactive specific transcript
