# Supplementary material for: BmncRNAdb: a comprehensive database of non-coding RNAs in the silkworm, Bombyx mori
Source: BMC Bioinformatics. 2016 Sep 13;17(1):370. doi: 10.1186/s12859-016-1251-y (PMC5022206; doi:10.1186/s12859-016-1251-y)
Supplement: Additional file 1: Table S1. — The detail information of RNA-seq datasets. All the samples used to the identification of the silkworm lncRNAs. (DOC 44 kb) [file 12859_2016_1251_MOESM1_ESM.doc]

**Table S1. Detail information of RNA-seq datasets**

| No | SRA accession | Number of samples | Tissue/Cell | Layout | Size (Gb) | reads length (bp) | Reference |
| --- | --- | --- | --- | --- | --- | --- | --- |
| 1. | SRP049193 | 2 | tissues | pair-end | 12.2 | 102 | [65] |
| 2. | DRP002360 | 2 | cells | pair-end | 14.7 | 101 | [64] |
| 3. | DRP000651 | 2 | cells | MatePair | 24.7 | 101 | / |
| 4. | DRP002346 | 2 | tissues | single-end | 3.2 | 36 | / |
| 5. | DRP001154 | 8 | tissues | pair-end | 30.7 | 101 | [63] |
| 6. | DRP001409 | 4 | tissues | pair-end | 16.2 | 100 | [63] |
| 7. | SRP038718 | 2 | tissues | pair-end | 10.2 | 101 | [62] |
| 8. | SRP038719 | 10 | tissues | single-end | 3.6 | 49 | [61] |
| 9. | SRP002417 | 1 | tissues | single-end | 0.31 | 36 | [60] |
| 10. | SRP026709 | 4 | tissues | pair-end | 11.5 | 100 | [59] |
| 11. | SRP008285 | 1 | tissues | pair-end | 2.8 | 75 | [58] |
| 12 | SRP007541 | 1 | tissues | single-end | 1.2 | 75 | [57] |
| 13 | SRP014173 | 2 | cell | single-end | 36.3 | 101 | [56] |
| 14 | SRP048500 | 4 | tissues | pair-end | 23.6 | 100 | [55] |
